# Squirrels reduce post-fire regeneration potential in serotinous pines

**DOI:** 10.1093/aob/mcag200

**Published:** 2026-07-09

**Authors:** Carmen Guiote, Eduardo T Mezquida, Juli G Pausas

**Affiliations:** Departamento de Ecología y Cambio Global. Centro de Investigaciones sobre Desertificación (CIDE, CSIC-UV-GV), Moncada, Valencia E-46113, Spain; Department of Ecology, Faculty of Sciences, Autonomous University of Madrid, Madrid 28049 , Spain; Biodiversity and Global Change Research Center (CIBC-UAM), Autonomous University of Madrid, Madrid 28049, Spain; Departamento de Ecología y Cambio Global. Centro de Investigaciones sobre Desertificación (CIDE, CSIC-UV-GV), Moncada, Valencia E-46113, Spain

**Keywords:** Pre-dispersal seed predation, fire traits, evolutionary ecology, *Pinus halepensis*, *Sciurus vulgaris*

## Abstract

**Background and Aims:**

Serotiny is a key trait for population persistence in fire-prone ecosystems, allowing species to accumulate an aerial seed bank until fire triggers seed release. However, serotinous cones also provide a food source for specialized pre-dispersal seed predators, potentially counteracting the benefits of serotiny. These predators may also exert selection pressures on cone traits that can further reduce the size of the canopy seed bank. Here we evaluate the mechanisms by which seed predation reduces the size of the canopy seed bank and quantify the magnitude of these effects.

**Methods:**

We sampled six sites dominated by mature *Pinus halepensis* forests, three with European red squirrels, *Sciurus vulgaris*, and three without. For each tree, we quantified cone production, determined the degree of serotiny, assessed seed predation (in squirrel-present populations) and characterized cones and seeds. This allowed us to estimate the size of the seed bank and the effects of seed predation on it. We also conducted a literature review for further validation.

**Key Results:**

Our findings indicate that, in populations with squirrels, pre-dispersal seed predation reduces the canopy seed bank by ∼66 %, not only through the ecological effect of cone removal, but also by exerting negative selection on serotiny and promoting increased seed defences.

**Conclusions:**

Pre-dispersal seed predation may compromise the post-fire regeneration of serotinous species by reducing the canopy seed bank, which is particularly concerning in the context of increasing fire activity in the Mediterranean basin.

## INTRODUCTION

Fire has been part of the natural dynamics of many ecosystems for millions of years (Pausas and Keeley, 2009). Therefore, plant species inhabiting fire-prone environments have developed traits that allow them to survive or recruit after fires (Keeley and Pausas, 2022). One of the most recognized traits associated with fire is serotiny, which is the accumulation of a canopy seed bank in woody structures (cones or fruits) until the heat of a fire opens them, releasing the seeds (Lamont *et al.*, 1991*a*, 2020). In environments where return intervals of crown fires fall between the age of reproductive maturity and the plant lifespan, serotiny maximizes the amount of seeds available when recruitment conditions are optimal, i.e. post-fire, as resource availability is high and competition is low (Causley *et al.*, 2016; Lamont *et al.*, 2020; best-bet strategy *sensu*Pausas *et al.*, 2022). Therefore, variations in serotiny can influence the structure and dynamics of ecosystems, making it essential to understand the factors determining its prevalence.

Even though it is well known that fire regimes shape intraspecific serotiny variability in many species and regions (i.e. Cowling and Lamont, 1985; Gauthier *et al.*, 1996; Groom and Lamont, 2011; Hernández-Serrano *et al.*, 2013; Lamont *et al.*, 2020), there is still considerable variation in serotiny within populations and among populations with similar fire regimes (Muir and Lotan, 1985; Benkman and Siepielski, 2004; Tapias *et al.*, 2004; Hernández-Serrano *et al.*, 2013). This indicates that factors other than fire likely contribute to shaping this trait. One disadvantage of storing a canopy seed bank is that seeds become a year-round available food source to seed predators (Lamont *et al.*, 1991*b*; Talluto and Benkman, 2014). This increased exposure of cones may increase pre-dispersal seed predation and thus reduce tree fitness; that is, predation can select against serotiny (Benkman and Siepielski, 2004; Talluto and Benkman, 2013). Therefore, the spatial variability in serotiny could be mainly due to the strength of these two opposing selective forces, fire and pre-dispersal seed predation (Talluto and Benkman, 2013, 2014). In short, although we have plenty of evidence that fire regimes shape serotiny, the potential of biotic interactions, such as seed predation, influencing the degree of serotiny has received limited attention.

Pre-dispersal seed predators also influence the evolution of pine cone characteristics, making them more resistant to being eaten (Smith, 1970, 1975; Benkman, 1995; Siepielski and Benkman, 2007; Parker and Benkman, 2020). This greater investment in defensive structures implies a reduction in the relative seed production per cone (Smith, 1970; Benkman, 1999; Mezquida and Benkman, 2005; Parker and Benkman, 2020), contributing to reducing the seed bank and thus potentially post-fire pine regeneration (Siepielski and Benkman, 2008; Talluto and Benkman, 2014). Although this cost should be particularly significant for serotinous species (as they primarily depend on their stored canopy seed bank for persistence), little is known about the combined impact of reduced serotiny and fewer seeds per cone as a result of seed predation.

In this study, we aim to understand the implications of pre-dispersal seed predation on post-fire regeneration potential in a serotinous pine. We hypothesize that pressure exerted by seed predators reduces the canopy seed bank and consequently the post-fire regeneration potential of populations, and that this reduction is mediated by (1) the direct ecological effect of cone removal (and therefore seeds); (2) negative selection against serotiny, which reduces the frequency of highly serotinous individuals in populations; and (3) an increase in seed defences, which leads to a decrease in the number of seeds per cone. We tested this hypothesis in the iconic and widespread Mediterranean pine *Pinus halepensis* (Aleppo pine), a species whose reproduction is tied to the fire regime (Hernández-Serrano *et al.*, 2013; Guiote and Pausas, 2023), and its major pre-dispersal seed predator, *Sciurus vulgaris* (European red squirrel; see more details about the studied species in Supplementary Data Notes S1). We first explored, based on extensive field sampling, the mechanisms that can lead to a reduction in the canopy seed bank by analysing the effects of defensive traits of pine cones, serotiny degree and crop size on predation pressure at the level of individual trees (within a population; Fig. 1A). Then we verified whether the patterns found are consistent in explaining the variation in seed defences, serotiny degree and size of the canopy seed bank among populations by comparing populations under contrasting seed predation pressures (regional analysis; Fig. 1B). Finally, we validated the relationship between serotiny and squirrels with an independent dataset. Specifically, we searched the literature for estimates of serotiny and combined them with records of squirrel presence in *P. halepensis* forests across the Mediterranean basin (geographical analysis; Fig. 1C).

**Fig. 1.**
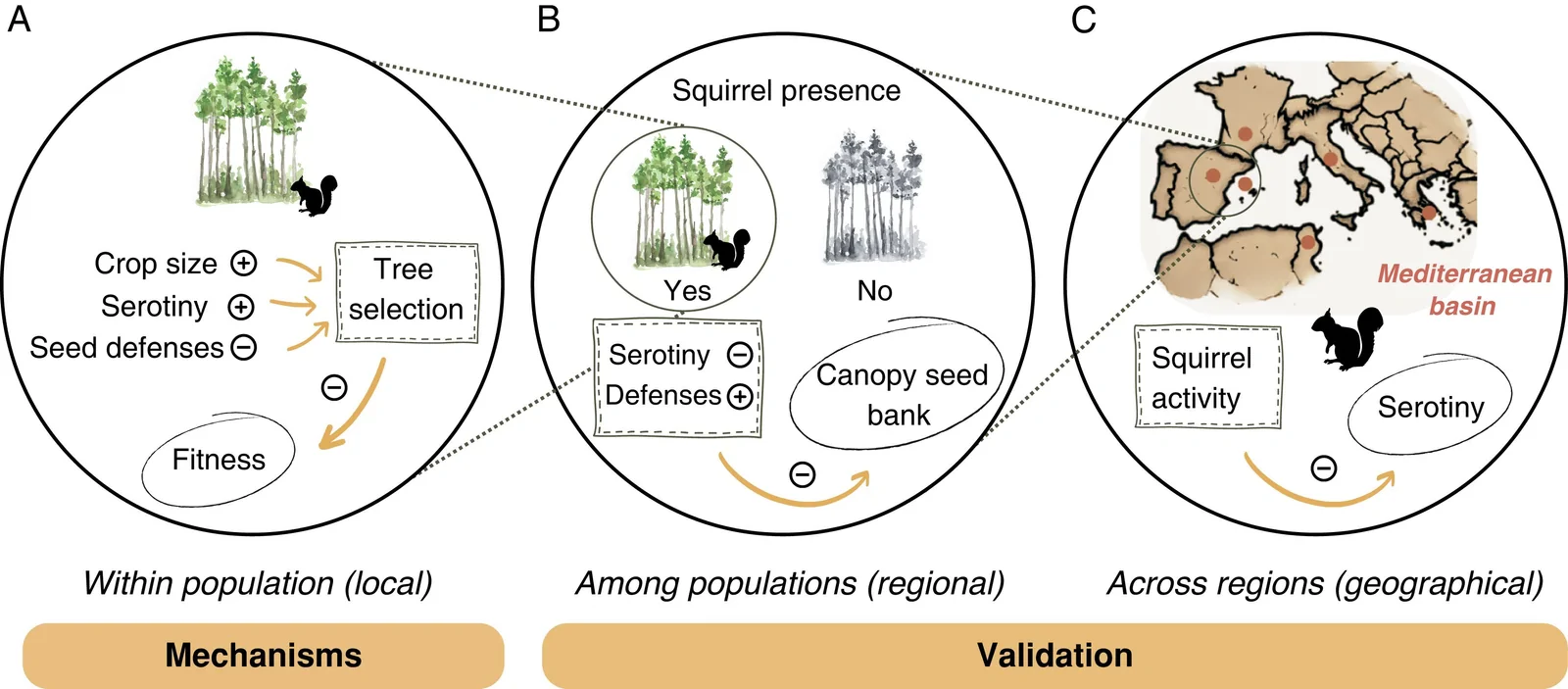
Conceptual diagram illustrating the working hypothesis about the impact of seed predation by squirrels on the size of the canopy seed bank in a serotinous pine species. (A) Trees within a population with larger crop sizes, higher serotiny levels and weaker seed defences are more likely to be preferentially harvested by squirrels, leading to a reduction in tree fitness (local scale). (B, C) This selection pressure is reflected in lower serotiny levels (validated at regional and geographical scales) and increased seed defences (validated at the regional scale; B) in populations where squirrels are present (B) or where their density is higher (C).

## MATERIALS AND METHODS

### Field sampling

We selected six sites dominated by mature *Pinus halepensis* forests in two contrasting seed predation environments. In three of these sites squirrels were absent. Two were located in Ibiza, a continental island (in the Balearic Archipelago) ∼90 km from the Iberian Peninsula; in fact, it is an extreme of the Baetic mountains of the Iberian Peninsula. The third was in a relatively isolated population on the Iberian Peninsula. In contrast, squirrels were present (although at different densities) at the other three sites, all located on the Iberian Peninsula (Supplementary Data Table S1). Site selection was based on distribution maps (www.vertebradosibericos.org; www.bdb.gva.es) and field observations of remains of consumed cones (cone cores) on the ground. We ensured that cone cores resulted from the feeding activity of squirrels rather than other vertebrate seed predators (such as rats or crossbills; following Rima *et al.*, 2007). The current presence of squirrels in Iberian *P. halepensis* forests likely results from both a long-standing ecological association (dating back at least to the Middle Ages) and more recent recolonization following the forests’ 20th-century regeneration. On the other hand, the historical absence of squirrels in the Balearic Islands is well documented (e.g. lack of fossil records), and thus it provides a unique opportunity for comparing the effects of the presence and absence of squirrels in relatively close distance and under similar conditions. Identifying regions in the Iberian Peninsula where squirrels were absent was more challenging, as squirrels are very common and the available information is scarce (Rocha *et al.*, 2014). However, squirrels depend on habitat quality and connectivity, so we located a relatively isolated *P. halepensis* population within the peninsula where squirrels were absent.

To minimize the effect of fire regime (Hernández-Serrano *et al.*, 2013, 2014) and tree age (Moya *et al.*, 2008; Martín-Sanz *et al.*, 2016) on serotiny degree, all populations were located in sites with a similar fire regime (relatively frequent crown fires) and in mature stands with similar tree sizes (Supplementary Data Table S2). However, the age of the trees and variations in fire regimes were considered in the analyses (see below). The selection procedure was based on the available fire maps, field evidence, and knowledge about the local relation between fire activity, altitude and summer drought (Verdú and Pausas, 2007; Hernández-Serrano *et al.*, 2013).

In the six sites we haphazardly selected pine trees (separated by at least 10 m, excluding trees with overlapping crowns) and measured their size (diameter at breast height; DBH) and the distances to the two closest conspecific trees (we used the mean of these values; hereafter average distance). Next, using binoculars, we counted the number of cones from the last six annual cone cohorts and recorded whether they were open or closed (closed cones older than 6 years are uncommon in this pine species; Hernández-Serrano *et al.*, 2013; Supplementary Data Notes S2). For each tree, we estimated serotiny degree, predation pressure (in sites with squirrels) and the crop size for each cohort (Table 1, Supplementary Data Notes S2). Finally, we collected at least six cones per tree from the most recent mature cohort, while avoiding malformed or damaged cones. For this, we climbed the pine crown and cut at least three terminal branches to represent the variability in cone traits within individuals.

**Table 1.** Definition of the variables used in this study.

Variable	Definition
Pre-predation serotiny degree	(Closed grey cones in the canopy + grey cone cores at the base of the pine)/(closed and open grey cones in the canopy + grey cone cores at the base of the pine)
Post-predation serotiny degree	Closed grey cones in the canopy/closed and open grey cones in the canopy
Predation pressure^a^	Cone cores at the base of the pine/(closed cones in the canopy + cone cores at the base of the pine)
Relative predation pressure	Predation pressure of the pine/mean value of predation pressure of the population
Crop size^a^	Closed cones + cone cores at the base of the pine
Canopy seed bank	Crop size × mean number of seeds per cone of the tree

^a^Cones were classified by cohorts (both in the canopy and at the base of the pine) as defined in the text.

In each site, 29 or 30 trees were sampled during spring (Supplementary Data Table S1). One of the sites with a high density of squirrels (Serra; the focal site) was selected to explore the influences of seed defences, serotiny degree and crop size on predation pressure at the individual level (local scale; Fig. 1A). For a squirrel, the rewards offered by a given tree often vary seasonally. For instance, a tree might produce numerous cones, making it highly profitable in spring, but it could be poorly serotinous and therefore less rewarding during autumn and winter. Thus, in this site we sampled also during winter (77 trees), when the ripened cones have passed the critical period (summer) and some have opened. Consequently, in this focal site we collected a minimum of six cones per tree in each sampling season.

### Cone trait measurements

Cones of all six sites were taken to the laboratory for measuring morphological traits following the methodology in Mezquida and Benkman (2005; see more details in Supplementary Data Notes S3). Additionally, we monitored the time required for each cone to open (in an oven; hereinafter ‘cone opening duration’), measured its width 45 minutes after opening (see detailed protocol in Supplementary Data Notes S3), and calculated the difference between the width of the open and closed cone divided by the width of the closed cone as an estimate of scale rigidity (a potential defensive trait; Supplementary Data Notes S3). As in previous studies (e.g. Mezquida and Benkman, 2005; Parker and Benkman, 2020), we also estimated the seed-to-cone mass ratio as the total seed mass (the number of full seeds, i.e. excluding empty seeds, multiplied by the mean mass of individual seeds) divided by the total cone mass. This variable is considered a defensive trait in such a way that lower levels of this ratio indicate higher defence value as squirrels need to chew more woody tissue relative to the energy intake (lower feeding efficiency; Smith, 1970; Benkman *et al.*, 2001, 2003, 2010; Mezquida and Benkman, 2005). Finally, we calculated mean values per tree for all measured traits to be used in the statistical analyses.

### Bibliographic review (geographical-scale analysis)

We searched the literature for articles where serotiny had been estimated in *P. halepensis*. We found four articles with data for 61 populations distributed in six geographical regions throughout the Mediterranean basin (Supplementary Data Table S3). To test the influence of predation, we used georeferenced data on *S. vulgaris* presence obtained from the GBIF database (GBIF.org, 2024). Then, using QGIS version 3.16.13 (QGIS Development Team, 2021), we set an influence area of 0.1° (∼10 km) radius around the studied populations and quantified squirrel record density per square kilometre as a proxy for squirrel occurrence and activity (hereafter, ‘squirrel activity’; Supplementary Data Table S3). In all the studies considered, the serotiny degree was estimated following the same criteria (Supplementary Data Notes S4).

### Statistical analyses

Climatic variables were obtained from the CHELSA v.2 database (a gridded map with 0.0083° resolution and average values for the 1981–2010 period; Karger *et al.*, 2021). Statistical analyses were performed using R software version 4.3.1 (R Core Team, 2023). Generalized linear mixed models (GLMMs) and zero-inflated γ models (ZIGs) were fitted using the glmmTMB package (Brooks *et al.*, 2017) while linear mixed models (LMMs) were fitted using the lme4 package (Bates *et al.*, 2015). Models were constructed through the sequential addition of variables, using Akaike’s information criterion (AIC) to guide model selection. The DHARMa package (Hartig, 2022) was used to assess model diagnostics via residual analysis, and the ggeffects package (Lüdecke, 2018) was used to compute predicted values. The main statistical analyses conducted in this study (excluding alternative model specifications for robustness checks and complementary analyses) are summarized in Table 2.

**Table 2.** Summary of the main statistical analyses performed in this study, including analytical objectives, model type, dependent variable, predictors and random effect.

Objective	Model^a^	Dependent variable	Predictors	Random effects
(A) Within a population				
To determine factors conditioning tree predation	ZIG	Relative predation pressure of the trees	Zero part: crop size of the target cohort	
			Conditional part: cone traits, pre-predation serotiny degree and their interactions	
(B) Among populations				
To test the influence of squirrel presence on serotiny degree	GLMM	Post-predation serotiny degree of the oldest cone cohorts considered	Squirrel presence, fire regime, tree size and density	Site
To identify selection pressures of predation on the canopy seed bank	LMM	Seed-to-cone mass ratio	Squirrel presence, climatic variables (precipitation and humidity), tree size and density	Site
	LMM	Total number of seeds per cone	Squirrel presence, cone length, scale thickness, climatic variables (precipitation and humidity), tree size and density	Site
To corroborate the impact of predation pressure on the canopy seed bank and estimate its magnitude	GLMM	Size of the canopy seed bank	Annual cone production and squirrel presence	Site
To assess whether the serotiny–seed defence relationship varied with predation pressure	LMM	Post-predation serotiny degree of the oldest cone cohorts considered	Interaction between serotiny categorization (stratification factor), seed-to-cone mass ratio and squirrel presence	Site
(C) Across regions				
To test the influence of predation pressure on serotiny across the Mediterranean basin	LMM	Serotiny degree	Squirrel activity, fire proneness and tree height	Site nested within study

^a^ ZIG (Zero-inflated γ), GLMM (Generalized linear mixed), LMM (Linear mixed).

#### Within a population (local-scale analysis)

In the focal site (Serra) we found that in winter (first sampling) most of the predated cones were serotinous (94.8 % of grey cones), while in summer (second sampling) most predated cones corresponded to the first mature cone cohort (81.1 % of orangish-brown cones). Therefore, we studied seed predation by fitting a model for each sampling period. To identify the most important variables in explaining relative predation pressure at the tree level, we fitted a ZIG model with the log link function. In the zero-inflated part of the model (i.e. probability of lack of seed predation), we included crop size of the target cohort in each sampling (grey cones in the first case, orangish-brown cones in the second), as it is crucial in the selection of individual plants by seed predators (Jordano, 2000). In the conditional part (i.e. predation pressure in predated trees), we included cone traits, pre-predation serotiny degree and their interactions. Additionally, we performed pairwise linear regressions between the relative predation pressure and cone traits or serotiny degree (values standardized to a mean of 0 and a variance of 1).

#### Among populations (regional-scale analysis)

We tested whether serotiny at the individual level differed among the six sites depending on squirrel presence using a GLMM with a β-binomial error distribution and the logit link function. Serotiny can be characterized based on two components; the proportion of serotinous cones and the time these cones remain closed (temporal component of serotiny; Lamont *et al*., 2020). We calculated post-predation serotiny degree (Table 1) using the two oldest studied cone cohorts for these analyses because it is the estimation that best reflects the temporal component of serotiny. In any case, we also made the analysis with all serotinous cone cohorts together. We included squirrel presence as predictor and site as a random factor. We also included proxies for the most relevant factors explaining serotiny in this species: fire regime (as fire proneness), tree size (as DBH) and density effects (as average distances to the two closest trees; Goubitz *et al*.,2004). In Mediterranean conditions low summer precipitation is often related to high fire-proneness (Keeley *et al.*, 2012) and, specifically in our study area, it is correlated with the area burned (Pausas, 2004; Pausas and Paula, 2012). Thus, we used the mean monthly precipitation of the warmest quarter as a proxy for fire proneness. We also considered an alternative proxy, the cumulative burned area over 37 years within a 10-km buffer around the sites (following Guiote and Pausas, 2023).

To investigate the selection pressures of predation on the canopy seed bank by the increase in seed defences, we began by testing whether the seed-to-cone mass ratio differed between populations with and without squirrels. This ratio has previously been identified as a defensive trait against squirrels and is known to correlate with the number of seeds per cone (Mezquida and Benkman, 2005; Parker and Benkman, 2020). Next, we examined whether the number of seeds per cone was different depending on squirrel presence. To do this, we fitted two LMMs in which the response variables were the seed-to-cone mass ratio and the total number of seeds per cone. In both we included squirrel presence as predictor and site as random factor. The size of the cone and the thickness of the scales are traits related to the amount of seeds (Mezquida and Benkman, 2005), so we also included cone length (highly correlated with width; *r* = 0.73, *P* = <0.001) and scale thickness when modelling the number of seeds per cone. Also, since seed production can be conditioned by factors such as water availability and intraspecific competition (Moya *et al.*, 2007; Ayari *et al.*, 2011), we tested a set of basic temperature- and precipitation-related variables (Supplementary Data Table S4) in addition to tree size (DBH) and the average distance to the closest trees. We excluded from these analyses cones with missing values for any of the traits (<6 % for seed-to-cone mass ratio and <1 % for the number of seeds per cone). Since we used tree mean values, we excluded trees with fewer than three cones with data for seed-to-cone mass ratio (11 cases) and trees with fewer than three cones with data for the amount of seeds per cone (5 cases). Additionally, we checked whether the other cone traits measured differed among sites with and without squirrels by fitting LMMs. We use each cone trait as a response variable, squirrel presence as predictor and site as random factor.

To corroborate the impact of predation pressure on the canopy seed bank (Table 1) and to estimate its magnitude, we tested whether the size of the canopy seed bank differs among populations depending on squirrel presence. For this, we fitted a GLMM with a γ error distribution and the log link function. We included annual cone production (crop size of the more recent mature cone cohort; in Serra we considered cone production from the spring sampling, as sampling at the other sites was conducted during spring) and squirrel presence as predictor variables, and site as a random factor. In this case we considered not only serotinous cones, since mature cones from more recent cohorts are also predated by squirrels and the seeds from these cones contribute to a certain extent to post-fire regeneration (Lamont and Enright, 2000; Greene *et al.*, 2024). Additionally, to assess how predation pressure impacts each of the two components of the canopy seed bank (number of cones and number of seeds per cone), we fitted two models with the same structure and error distribution family as our previous analysis. In this case, to estimate the canopy seed bank, in the first model we held the number of seeds per cone constant at its mean value, while in the second model, we kept the number of cones constant at its mean value. We then determined the effect size of predation pressure on each trait by calculating Cohen's *d* between the levels of squirrel presence (No, Yes) in both models.

Finally, we examined whether the association between serotiny and seed-to-cone mass ratio (i.e. seed defences) differed between populations with and without squirrels. Finding a stronger association at sites with squirrels could indicate that both traits are being jointly selected due to predation, potentially counteracting the negative effects of seed predation on serotiny (Parker and Benkman, 2020). In pine species where serotiny is a qualitative trait (i.e. individuals produce predominantly serotinous or non-serotinous cones), such association has been noted for serotinous individuals within a population (Parker and Benkman, 2020). Therefore, since serotiny is a quantitative trait in our species (Nathan *et al.*, 1999), a stronger association might only be evident in the most serotinous individuals of the population. For this reason we categorized trees as strong versus intermediate-weak serotinous (above and below the 75th percentile, respectively). We considered the interaction between this categorization, the seed-to-cone mass ratio and squirrel presence as predictors in the model. Note that this categorization was used as a stratification factor to test whether the slope of the relationship between serotiny and seed-to-cone mass ratio differed between serotiny classes. We fitted an LMM with post-predation serotiny degree (arcsine-transformed) as the response variable and site as a random factor. This analysis was also performed using a ZIG model rather than the arcsine transformation, but the poor fitting (deviation from residual normality) made us use this transformation (no deviation of the residuals). Before constructing the model, we performed an exploratory analysis to assess potential relationships between serotiny and cone traits across all populations. This initial analysis identified covariates potentially associated with serotiny (independent of seed predation pressure by squirrels) for inclusion in the main model, enhancing its accuracy and accounting for possible confounding factors.

#### Across regions (geographical-scale analysis)

Using the bibliographic review data, we tested the influence of predation pressure on serotiny across the Mediterranean basin. To do so, we fitted an LMM including serotiny degree (proportion of closed cones relative to total cones, with the two youngest cone cohorts excluded) as a response variable, squirrel activity as a predictor variable, and site nested within study as random factor. As in the regional analyses, we also took into account factors that can affect serotiny degree: fire proneness (as an indicator of fire regime) and the height of the trees. We use the arcsine square-root transformation of serotiny to fit the model.

## RESULTS

### Within population (local-scale analysis)

Crop size significantly affected the relative predation pressure in both sampling seasons. The smaller the crop size of a tree, the lower the probability of being predated. This is indicated by the negative relation between the probability of not being predated and crop size in the zero-inflation part of the models (Table 3.1A, 3.2A). The conditional part of the model for the winter sampling indicated that the most serotinous trees with a higher seed-to-cone mass ratio experienced the highest relative predation in winter (Table 3.1B, Fig. 2A). During spring, scale rigidity was the most relevant variable in explaining the differences in relative predation pressure among trees, with trees having cones with softer scales being preferred by squirrels (Table 3.2B, Fig. 2B).

**Fig. 2.**
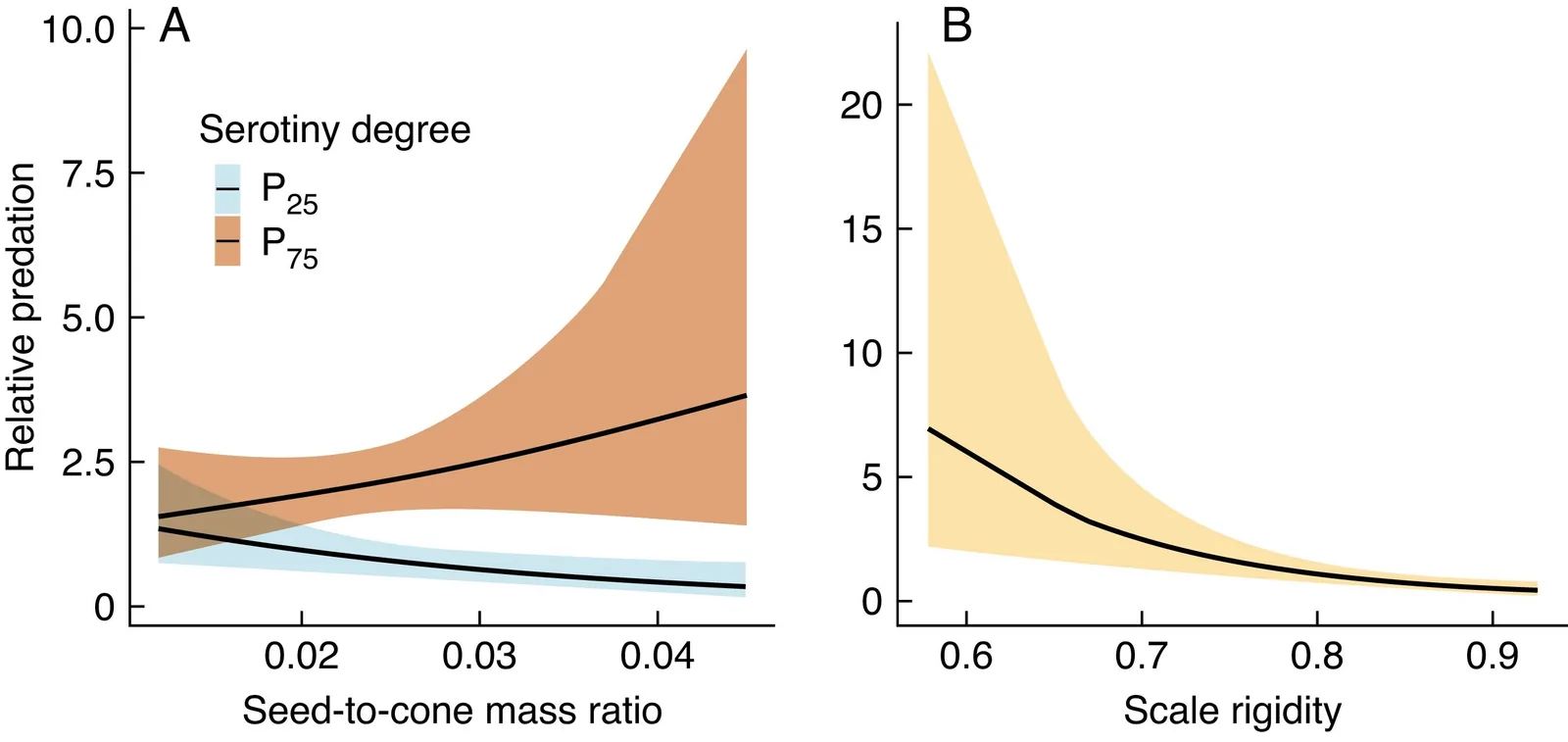
Fitted lines of the models testing the effects on trees’ relative predation pressure against (A) the interaction between serotiny degree and seed-to-cone mass ratio in winter, and (B) scale rigidity in spring. For the former (A), we provide fitted lines for the 25th (P_25_, blue) and 75th (P_75_, orange) percentiles of serotiny. Lower values of seed-to-cone mass ratio and higher values of scale rigidity are associated with enhanced seed defences as they reduce feeding efficiency for the squirrels. See Table 3 for the statistics.

**Table 3.** Summary of the zero-inflated γ (ZIG) models testing the factors determining relative predation pressure within a population (across individual trees of the focal site) during winter (1) and spring (2). The models were fitted by sequential addition of variables. The last two columns provide the estimated coefficients and standard errors for the intercept (null model row) and fixed effects of the final model.

Model	d.f.	AIC	*χ* ^2^	*P*	Estimate	s.e.
(1) Winter sampling (ZIG)						
(A) Zero-inflation model						
Null	3	247.36	–	–	0.540	0.396
Crop size (grey cones)	4	240.70	8.66	0.003**	−0.023	0.009
(B) Conditional model						
Null	4	240.70	–	–	1.173	0.747
Serotiny	5	228.89	13.81	<0.001***	−1.232	1.216
+ Seed-to-cone mass ratio	6	230.40	0.49	0.483	−85.113	27.615
+ Serotiny × Seed-to-cone mass ratio	7	225.84	7.05	0.029*	136.685	47.550
(2) Spring sampling (ZIG)						
(A) Zero-inflation model						
Null	3	94.869	–	–	2.168	1.43
Crop size (orangish-brown cones)	4	88.487	8.38	0.004**	−0.086	0.039
(B) Conditional model						
Null	4	88.487	–	–	−1.586	0.541
Scale rigidity	5	*81.208*	9.28	0.002**	−8.313	2.50S

Significance levels: *P <* 0.05 (*), *P <* 0.01 (**), and *P <* 0.001 (***).

Pairwise relationships between relative predation pressure on the trees and cone traits, as well as serotiny degree, showed that, during the winter sampling, only serotiny had a positive effect on predation pressure. In spring, both scale rigidity and seed-to-cone mass ratio exhibited a correlation with relative predation (negative and positive, respectively; Supplementary Data Table S5).

### Among populations (regional analysis)

Tree size (DBH) was the main explanatory variable for serotiny degree of the trees (negative relationship; Table 4A). After including this variable, the presence of squirrels still showed a significant negative relationship with serotiny (Table 4A, Fig. 3A). When we considered all grey cone cohorts (instead of the oldest cohorts), the trend towards lower serotiny in populations with squirrels was maintained, although the effect became marginal (Supplementary Data Table S6). Fire regime was not significant, regardless of whether we considered climate-based fire-proneness or cumulative burned area (Supplementary Data Table S7).

**Fig. 3.**
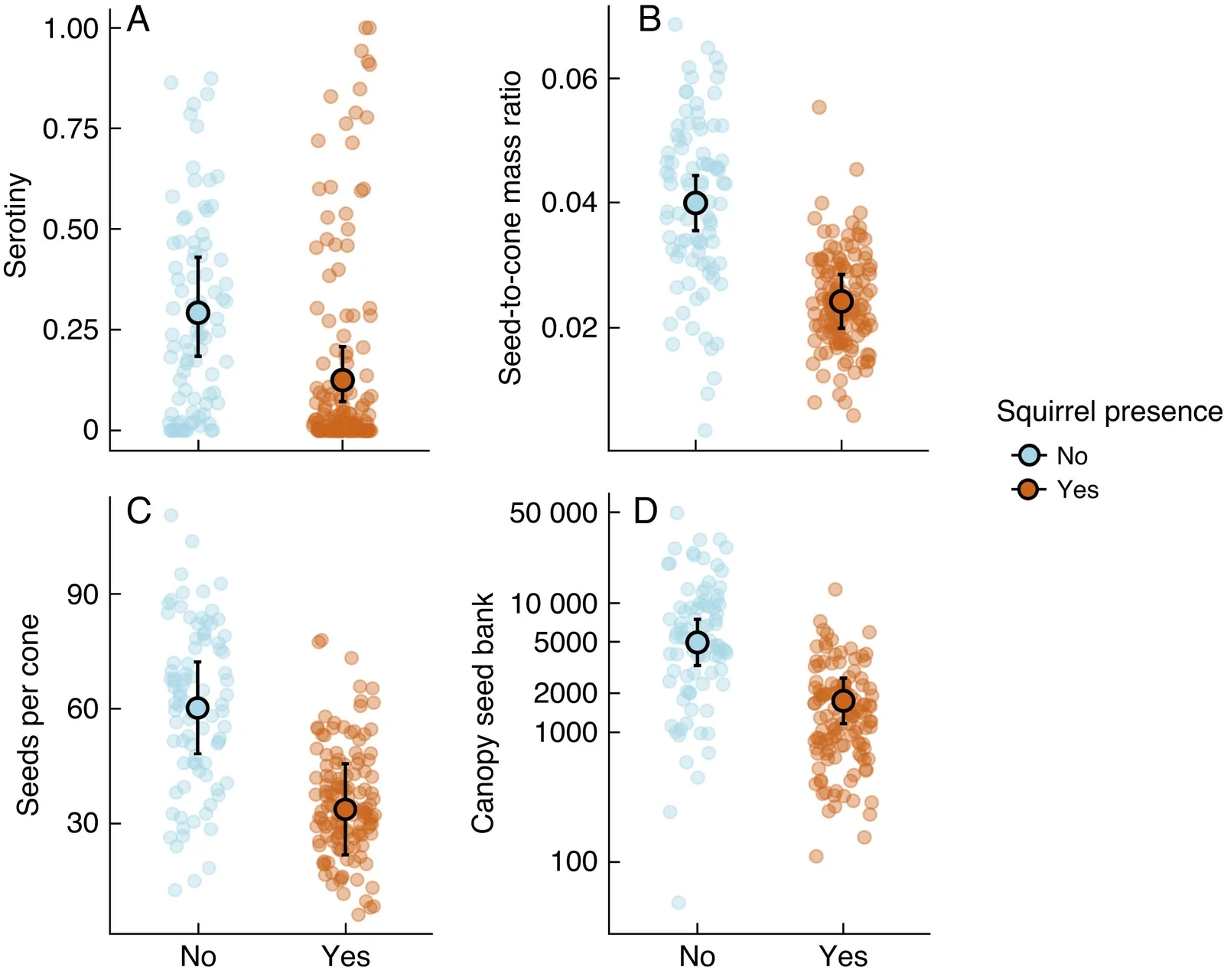
Comparison between absence and presence of squirrels (No, in blue; Yes, in orange, respectively) for serotiny degree (closed-to-total cones of the two oldest cone cohorts; A), seed-to-cone mass ratio (B), seeds per cone (C), and size of the canopy seed bank (log-scaled *y*-axis; D). Coloured symbols represent raw data per tree, while black-bordered coloured symbols with error bars indicate model-predicted values with 95% confidence intervals (see Table 4 for the statistics).

**Table 4.** Results of the model testing differences among populations with and without squirrels (squirrel’s presence) in: (A) serotiny, (B) seed-to-cone mass ratio (inverse of seed defences), (C) number of seeds per cone, and (D) size of the canopy seed bank (log-transformed). The models were fitted by sequential addition of variables. The last two columns provide the estimated coefficients and standard errors for the intercept (null model row) and fixed effects of the final model. Fitted values are shown in Fig. 3.

Model	d.f.	AIC	*χ* ^2^	*P*	Estimate	s.e.
(A) Serotiny (GLMM)						
Null	3	1368.7	–	–	0.861	0.596
DBH	4	1358.6	12.09	<0.001***	−0.065	0.018
+ Squirrel presence	5	1356.5	4.17	0.04*	−1.057	0.434
(B) Seed-to-cone mass ratio (LMM)						
Null	3	−1345.4	–	–	0.041	0.002
Squirrel presence	4	−1358.7	15.38	<0.001***	−0.016	0.002
(C) Number of seeds per cone (LMM)						
Null	3	1804.1	–	–	58.197	10.124
Cone length	4	1757.5	48.64	<0.001***	1.128	1.128
+ Scale thickness	5	1731.8	27.68	<0.001***	−18.158	3.321
+ Squirrel presence	6	1726.2	7.64	0.006**	−26.664	8.480
(D) Canopy seed bank (GLMM)						
Null	3	3902.7	–	–	8.285	0.247
Annual cone production	4	3856.1	48.58	<0.001***	0.015	0.002
Squirrel presence	5	3851.6	6.48	0.012*	−1.068	0.319

Asterisks indicate significance levels: *P <* 0.05 (*), *P <* 0.01 (**), and *P <* 0.001 (***).

There were significant differences in seed defences (seed-to-cone mass ratio) between populations with and without squirrels. Locations where predators were present had seed defence values that were ∼40 % higher on average (i.e. a lower seed-to-cone mass ratio) compared with those without predators (Table 4B, Fig. 3B). As expected, this resulted in cones with fewer seeds in populations with squirrels (on average, 43.3 % fewer seeds), after accounting for the variables related to the amount of seeds per cone (Table 4C, Fig. 3C). None of the environmental covariates tested (Supplementary Data Table S4) were related to the number of seeds per cone, and no other cone traits differed between populations with and without squirrels (Supplementary Data Table S8).

The size of the canopy seed bank was significantly lower in sites with squirrels (66 % less; Table 4D, Fig. 3D). Specifically, seed predation appeared to have a greater impact on the number of cones than on the number of seeds per cone (65.6 and 34.4 % of the total reduction, respectively; Supplementary Data Table S9, Supplementary Data Fig. S3).

Of all the cone traits measured, cone opening duration and scale rigidity were the only traits associated with serotiny across all populations (Supplementary Data Table S10). When we considered the effect of cone opening duration, scale rigidity was no longer significant (Supplementary Data Table S11). In populations where squirrels were present, serotiny was negatively related to seed-to-cone mass ratio (i.e. positively related to seed defences) only in the strongly serotinous trees (above the 75th percentile; Table 5, Fig. 4). This analysis was also performed using a zero-inflated binomial approach (instead of the arcsine-square root transformation), and even though the residuals deviated from normality (Kolmogorov–Smirnov normality test, *P* = 0.01) the results were the same (Supplementary Data Table S12).

**Fig. 4.**
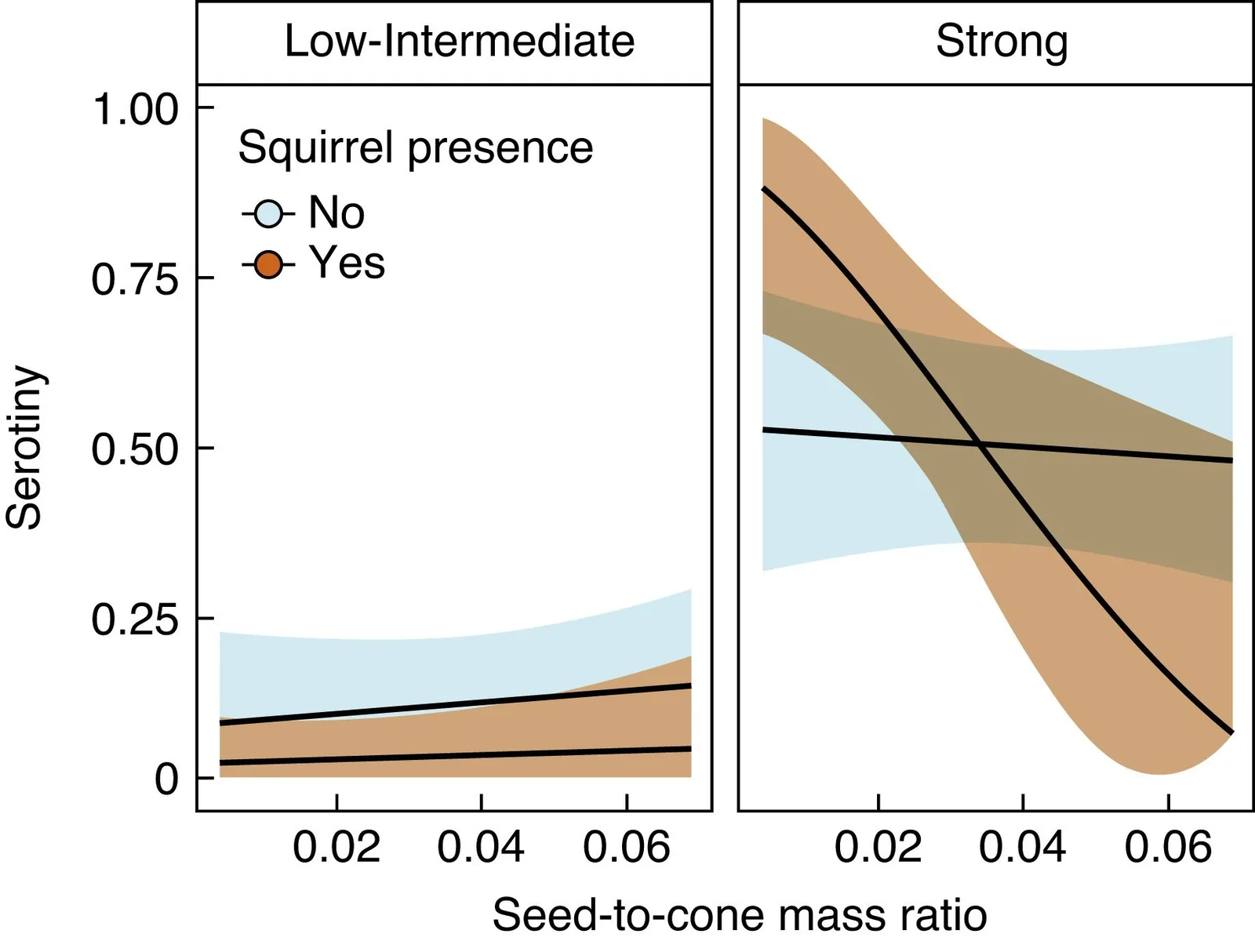
Fitted lines for the model testing the interaction between squirrel presence (No, in blue; Yes, in orange) and the serotiny categorization (low–intermediate vs strong) in explaining the association between serotiny degree and seed-to-cone mass ratio (inverse of seed defences). The left plot (Low-Intermediate) includes trees with serotiny degree below the 75th percentile, and the right plot (Strong) include those above this value. Serotiny values were back-transformed to generate the plot. The result of the model is shown in Table 5.

**Table 5.** Results of the LMM testing the association between serotiny (arcsine square root transformation) and seed-to-cone mass ratio in relation to squirrel presence.

Model	Estimate ± s.e.	*t* value	*P* value
Intercept	0.327 ± 0.070	4.653	0.007**
+ Cone opening duration	0.076 ± 0.015	5.181	<0.001***
+ Serotiny categorization	0.473 ± 0.057	8.356	<0.001***
+ Seed-to-cone mass ratio	0.021 ± 0.026	0.807	0.421
+ Squirrel presence	−0.156 ± 0.096	−1.620	0.179
+ Serotiny categorization × seed-to-cone mass ratio	−0.032 ± 0.040	−0.796	0.427
+ Serotiny categorization × squirrel presence	0.185 ± 0.081	2.284	0.023*
+ Seed-to-cone mass ratio × squirrel presence	−0.007 ± 0.038	−0.185	0.853
+ Serotiny categorization × seed-to-cone mass ratio × squirrel presence	−0.181 ± 0.090	−2.022	0.044*

Asterisks indicate significance levels: *P <* 0.05 (*), *P <* 0.01 (**), and *P <* 0.001 (***).

### Among regions (geographical-scale analysis)

Tree height and fire proneness were the most significant variables explaining the variability in serotiny degree among regions. Specifically, shorter trees and those from more fire-prone sites exhibited higher levels of serotiny. After accounting for these two factors, squirrel activity remained significant, demonstrating a negative relationship with serotiny (Table 6, Fig. 5), which is consistent with our regional analysis (Fig. 3A). As in the regional-scale analysis, the results using untransformed serotiny values (Supplementary Data Table S13) were similar, although this analysis exhibited a slight deviation from residual normality (Kolmogorov–Smirnov normality test, *P* = 0.01).

**Fig. 5.**
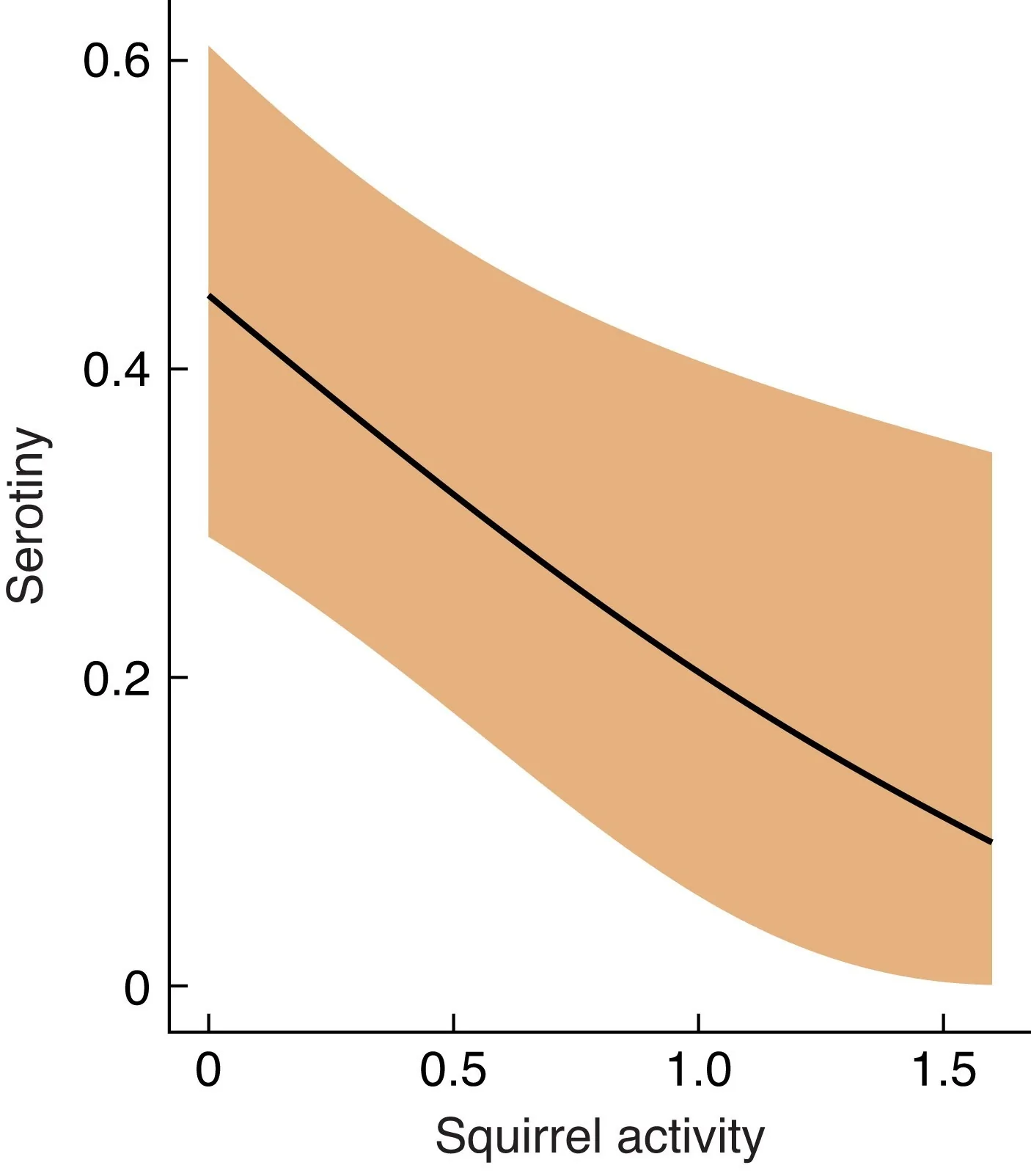
Fitted line of the model testing the influence of squirrel density (records per square kilometre of land) on serotiny variability (closed-to-total cones) across the Mediterranean basin. The model includes tree height and fire proneness as covariates (Table 6). The plot was generated using back-transformed serotiny values.

**Table 6.** Results of the model for serotiny variability among regions across the Mediterranean basin (serotiny values obtained from a literature search) in relation to tree height, fire proneness and squirrel activity. The models were fitted by sequential addition of variables. The last two columns provide the estimated coefficients and standard errors for the intercept (null model row) and fixed effects of the final model. The model is displayed in Fig. 6.

Model	d.f.	AIC	*χ* ^2^	*P*	Estimate	s.e.
Null	5	634.50	–	–	1.414	0.124
Tree height	6	586.07	50.43	<0.001***	−0.049	0.007
+ Fire proneness	7	571.48	16.59	<0.001***	0.004	0.001
+ Squirrel activity	8	568.55	4.93	0.026*	−0.234	0.104

Asterisks indicate significance levels: *P <* 0.05 (*), *P <* 0.01 (**), and *P <* 0.001 (***).

## DISCUSSION

Squirrels are voracious predators of pine seeds, and their direct and indirect effects contribute to a reduction of ∼66 % in the canopy seed bank of *P. halepensis.* This decline is driven by the ecological effects of cone removal, the stronger selection pressure on the most serotinous individuals, and the increased investment in seed defences by trees in response to predation at the expense of the number of seeds per cone (Fig. 6).

**Fig. 6.**
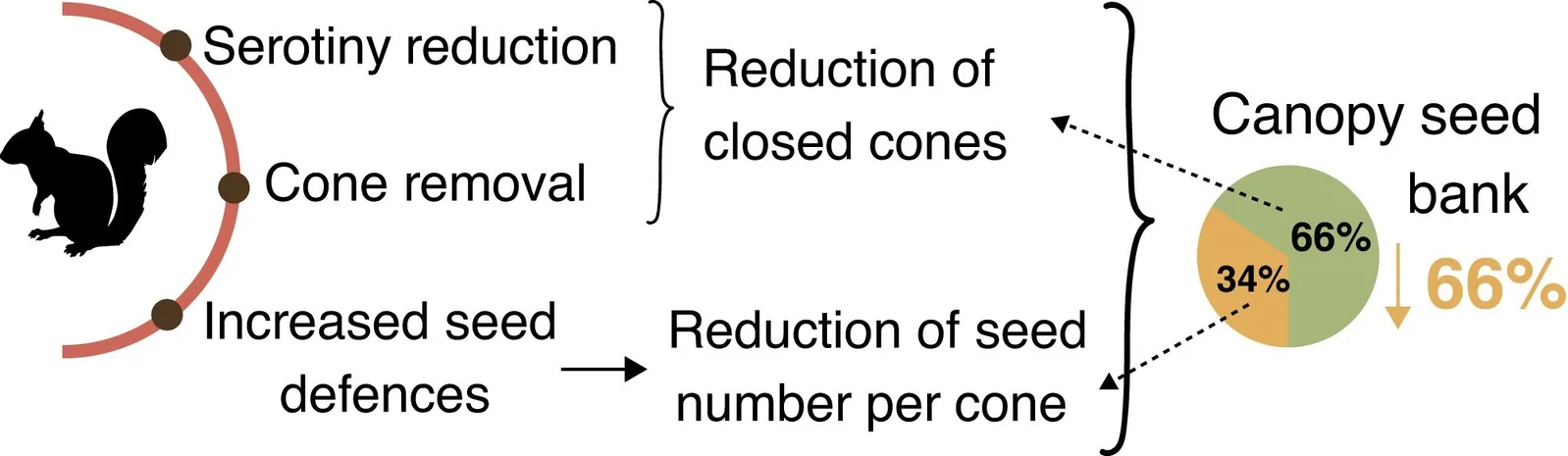
Conceptual diagram illustrating canopy seed bank reduction in *P. halepensis* populations. Predation by the European red squirrel reduces the canopy seed bank through two complementary mechanisms: (1) reduced serotiny and cone removal, leading to fewer closed cones per tree, and (2) selection favouring seed defences, leading to fewer seeds per cone. Together, these effects reduce the canopy seed bank by ∼66 %, with 66 % of the total reduction attributable to fewer closed cones and 34 % to fewer seeds per cone.

### Squirrels reduce serotiny level

For frugivorous and granivorous animals, although reward quality ultimately guides fine-scale foraging choices, resource abundance also plays a significant role in the overall decision-making process (Palacio *et al.*, 2016). This can explain why, during winter, when the availability of newly ripened cones is reduced due to seed predation in spring and summer, the presence of serotinous cones retained in the canopy becomes a key factor determining predation levels on individual trees, increasing predation pressure on the most serotinous trees within populations. The negative effect on serotiny was also detected at regional and geographical scales, indicating consistency across spatial scales and datasets. This pattern may simply reflect a direct ecological consequence of seed predation, namely the removal of serotinous cones by squirrels. However, serotiny is a key and heritable trait that increases fitness in fire-prone environments (Keeley and Zedler, 1998; Hernández-Serrano *et al.*, 2014; Castellanos *et al.*, 2015). Therefore, the increased predation pressure on the most serotinous individuals (at least during winter) likely reduces their relative fitness, resulting in a decreased frequency of highly serotinous individuals in the next generation (i.e. low serotiny is favoured). Considering this, it is reasonable to infer that among-population variation in serotiny levels (for given environmental conditions) results from both the ecological impact of seed predation and its evolutionary consequences.

### Pines protect their seeds against squirrels

Evidence for selection on seed-to-cone mass ratio during spring suggests that this trait may influence selection by squirrels during that season. However, its effect weakens when accounting for scale rigidity, likely because both traits contribute to feeding efficiency. Scale rigidity is associated with lignin content, which influences the hardness of scales (Smith, 1970; Moya *et al.*, 2008), with greater rigidity increasing the time and energy required by a squirrel to bite off scales to access the underlying seeds. Similarly, a lower ratio of seed to cone mass results in a higher proportion of woody tissue that squirrels must chew through to access a given amount of food (Benkman, 1999; Benkman *et al.*, 2001). Previous research indicates that squirrels prefer softer cones when available (e.g. Smith, 1970), and our spring sampling aligns with this pattern. However, we found no significant relationship between scale rigidity and predation pressure during the winter sampling. This absence of a relationship does not necessarily negate its role as a defensive trait, as it might result from reduced variability in this trait once the cones become serotinous (after summer), making them uniformly hard (Moya *et al.*, 2008; Greene *et al.*, 2024). The intrinsic positive association between serotiny and scale rigidity, combined with the role of this trait as a defence against seed predation during spring, suggests that being potentially more serotinous (i.e. having recently matured cones with more rigid scales) may serve a protective function against seed predation during certain periods. However, seed predation during spring is likely to have a relatively limited effect on post-fire regeneration (which represents the dominant regeneration strategy in this species). This is because tree selection by squirrels appears to be strongly driven by overall cone abundance. Consequently, trees producing large cone crops may be preferentially targeted in this season despite exhibiting cone traits associated with serotiny that confer partial resistance to predation. In this context, cone availability may outweigh the protective effect associated with serotinous cone characteristics. Moreover, because this species exhibits quantitative serotiny, in spring even highly serotinous trees retain a fraction of cones that will open during summer. As a result, spring predation is not restricted exclusively to serotinous cones. In contrast, winter predation is expected to have a stronger impact on fitness because all cones consumed during this season are serotinous, and they contribute the most to regeneration success since they perform better under post-fire environmental conditions (Goubitz *et al.*, 2002).

The observed increased investment in seed defences in populations with squirrels seems to be an evolutionary response to the negative selection of squirrels against trees with high seed-to-cone mass. Furthermore, the lower seed numbers observed in populations with squirrels suggest that selection favouring this defensive trait ultimately has a fitness cost at the population level. This reduction in seed number has previously been identified as a consequence of pre-dispersal seed predation in several conifer species (e.g. Mezquida and Benkman, 2005; Parker and Benkman, 2020). However, in contrast to previous findings in *P. halepensis* (Mezquida and Benkman, 2005), we did not find evidence of tree selection based on cone size or scale thickness. These contrasting results between studies may be due to differences in pine cone sizes between studies. Squirrels tend to select larger pine cones when the average cone size is relatively small, with this relationship reversing when cones are larger, within and among populations (Mezquida and Benkman, 2005; Siepielski and Benkman, 2007).

The pine cones in Serra (where selection by squirrels was measured) had an average length of 65.4 mm, which is smaller than the optimal cone length of ∼70 mm reported by Mezquida and Benkman (2005). Therefore, selective predation on the largest cones within the population could be expected; however, we found no evidence of such selection. Also, the average cone length reported for the sites with squirrels studied by Mezquida and Benkman (2005) was 75.79 mm, considerably larger than the mean cone length observed in our populations with squirrels (55.53 mm). Although we did not investigate the causes underlying these differences in cone size, they may help explain the absence of size-based selection in our populations. Whether the temporal disruption of the possible evolutionary trajectory towards an increase in cone size could be explained by, for example, the drought experienced by Mediterranean ecosystems in recent decades and its potential impact on cone and seed development (Moya *et al.*, 2007; Ayari *et al.*, 2011; Vogel *et al.*, 2021; IPCC, 2023) remains to be explored. An alternative explanation could be the contribution of a secondary pre-dispersal seed predator such as the crossbill (*Loxia curvirostra*) to the evolution of cone traits in our population (see Mezquida and Benkman (2005) for a geographic selection mosaic on cone traits influenced by the presence and abundance of crossbill and squirrels). That is, it is possible that on our sites that lacked or had a low density of squirrels, a secondary seed predator may have obscured the pattern of selection by squirrels on cone traits. In any case, as a response to predation pressure, populations with squirrels showed a lower number of seeds per cone, which may contribute to compromising post-fire regeneration of populations.

### Serotiny and seed defences association

In a *Pinus contorta* population, serotiny and seed defences showed a positive association, suggesting that the most serotinous individuals invest more in defences, counteracting the selection exerted by squirrels (*Tamiasciurus hudsonicus*) against serotiny (Parker and Benkman, 2020). In *P. halepensis*, the association between serotiny and seed-to-cone mass ratio in populations with squirrels is consistent with joint selection acting on both traits. Given that higher levels of predation lead to stronger selection (Benkman, 2013) and that squirrels prey upon the most serotinous trees during winter, it is therefore reasonable that defensive traits are enhanced in the upper range of the serotiny distribution within populations. Considering these results, together with the lower values of serotiny observed in sites with squirrels, we conclude that, although enhanced seed defences are especially favoured among highly serotinous individuals, they do not completely counteract the selective impact against serotiny by seed predators.

### Reduction of the canopy seed bank

Although *P. halepensis* may retain some capacity for regeneration via non-serotinous cones (Daskalakou and Thanos, 1996; Ne’eman *et al.*, 2004), the canopy seed bank remains the primary source of recruitment in Mediterranean fire-prone ecosystems (Goubitz *et al.*, 2002; Ne’eman *et al.*, 2004). So, while the 66 % reduction in *P. halepensis* may not be as extreme as in *Pinus contorta* populations in the Rocky Mountains, where American red squirrels are estimated to reduce the canopy seed bank by up to 95 %, severely limiting post-fire regeneration (Benkman, 2025) , it still represents a substantial loss of reproductive potential. This may be especially relevant under suboptimal post-fire conditions (e.g. drought, poor soils, or competition; Pausas *et al.*, 2002). Therefore, such a reduction could, in some situations, compromise forest recovery and the long-term persistence of populations.

### Conclusions

Serotinous species rely on canopy seed banks for post-fire recruitment, making it critical to understand the factors that regulate their dynamics. Increasing evidence suggests that climate change may lead to demographic collapse in these species (Vincenzi and Piotti, 2014; Enright *et al.*, 2015; Agne *et al.*, 2022; Souto-Veiga *et al.*, 2022; Paneghel *et al.*, 2024; Enright and Agne, 2025). Shorter fire intervals limit canopy seed bank development, while intensified drought conditions reduce the window for seedling emergence, delay reproductive maturity and decrease seed production. These factors, combined with the pre-dispersal seed predation, which have demographic and evolutionary consequences beyond the immediate loss of seeds (e.g. Parker and Benkman, 2020; Chiapero *et al.*, 2025), could severely threaten the future of populations in certain regions. This study therefore highlights the importance of closely monitoring serotinous populations in areas with abundant squirrels, as they could be especially susceptible to regeneration failure. Nevertheless, a reduced seed bank is not always detrimental; in certain Mediterranean areas historic management practices have resulted in overly dense pine stands that constrain natural regeneration of other species and biodiversity (Gómez-Aparicio *et al.*, 2009). Thus, incorporating knowledge of seed predation effects into management and restoration planning, taking into account site-specific and ecological objectives, may be crucial for sustaining ecosystem resilience under future climate scenarios.

## Supplementary Material

mcag200_Supplementary_Data

## SUPPLEMENTARY DATA

Supplementary data are available at *Annals of Botany* online. The document Supplementary Material provides all supplementary material cited in the text, which expands on the methodological details and includes tables with information on the additional minor analyses.

## AVAILABILITY OF DATA AND CODE

The data supporting this article are available at the following link: https://zenodo.org/records/21481506.

## AI ASSISTANCE

Artificial intelligence tools were used solely to improve the clarity and quality of the English writing.
