# Supplementary material for: Squirrels reduce post-fire regeneration potential in serotinous pines
Source: Ann Bot. 2026 Jul 9;138(5):1591–603. doi: 10.1093/aob/mcag200 (PMC13715016; doi:10.1093/aob/mcag200)
Supplement: Supplementary file 1 [file mcag200_supplementary_data.docx]

**Supplementary Material**

Original article: Guiote C, Mezquida E, Pausas JG. Squirrels reduce post-fire regeneration potential in serotinous pines. *Annals of Botan*y.

**Content:**

[**Table S1:** Details of the sampled sites.	2](#_Toc232949457)

[**Table S2:** Summary of models for a) DBH and b) fire proneness in relation to squirrel presence.	2](#_Toc232949458)

[**Notes S1:** Study species.	3](#_Toc232949459)

[**Notes S2:** Sampling procedure details.	3](#_Toc232949460)

[**Figure S1:** Differences in pine cone colour depending on their maturation stage.	4](#_Toc232949461)

[**Notes S3:** Cone trait measurement details.	4](#_Toc232949462)

[**Figure S2:** Schematic representation of scale traits measured on pine cones.	5](#_Toc232949463)

[**Table S3:** Details of the literature search study sites.	5](#_Toc232949464)

[**Notes S4:** Details about the methods to test the effect of predation on serotiny degree at geographical scale.	6](#_Toc232949465)

[**Table S4:** Climatic variables included in the model to test the influence of squirrel presence in explaining seed production per cone.	8](#_Toc232949466)

[**Table S5:** Summary of pairwise linear regression testing the influence of cone traits and serotiny in relation to predation degree in each Serra sampling; a) winter and b) spring).	8](#_Toc232949467)

[**Table S6:** Results of the model for serotiny degree considering all grey cone cohorts in relation to squirrels' presence and covariates.	9](#_Toc232949468)

[**Table S7:** Results of model for serotiny degree (mean values per site) in relation to cumulative burned area (CBA).	10](#_Toc232949469)

[**Table S8:** Mean cone measurements of the six sampled sites and summary of the models (linear mixed models including site as random factor) testing the influence of squirrel’s presence in each cone trait.	10](#_Toc232949470)

[**Table S9:** Summary of the models for a) canopy seed bank estimated holding the seed per cone constant, and b) canopy seed bank estimated holding the number of cones constant in relation to squirrel presence (considering annual crop size).	11](#_Toc232949471)

[**Figure S3:** Effect of squirrel’s presence on the two components of the canopy seed bank, cone number and seeds per cone.	11](#_Toc232949472)

[**Table S10:** Summary of pairwise linear regression testing the relationship of cone traits and serotiny (arcsine-transformed) across all populations.	12](#_Toc232949473)

[**Table S11:** Results of the model for serotiny degree (arcsine-transformed) in relation to cone traits (sequential addition of variables).	12](#_Toc232949474)

[**Table S12:** Results of the zero-inflated Gaussian mixed model testing the association between serotiny and seed-to-cone mass ratio in relation to squirrel presence.	13](#_Toc232949475)

[**Table S13:** Results of the of the zero-inflated Gaussian mixed model testing serotiny variability among regions across the Mediterranean basin (geographical analysis) in relation to tree height, fire proneness, and squirrel activity.	13](#_Toc232949476)

[**References**	14](#_Toc232949477)

Table S1: Details of the sampled sites. Locality, region (IB= Iberian Peninsula, BI= Balearic island), geographical coordinates (Coordinates; latitude and longitude; ^o^), number of sampled trees (N), squirrel presence (and density), diameter at breast height (DBH; cm), average distances to the closest trees (A.D, m), fire proneness as the mean monthly precipitation amount of the warmest quarter (F.P; kg m^-2^ month^-1^) and mean serotiny values of the sites (Serotiny; %).

| Locality | Region | Coordinates | N | Squirrels | DBH | A.D | F.P | Serotiny |
| --- | --- | --- | --- | --- | --- | --- | --- | --- |
| Serra | IB | 39.67, -0.49 | 77 + 30* | Yes (High**) | 22.89 | 2.95 | 55.2 | 25.46 |
| Artana | IB | 39.91, -0.25 | 30 | Yes (High**) | 28.40 | 3.57 | 77.5 | 5.09 |
| Vandellòs i l'Hospitalet de l'Infant | IB | 41.02, 0.89 | 30 | Yes (Low**) | 30.80 | 3.99 | 146 | 3.89 |
| Llombay | IB | 39.31, -0.57 | 30 | No | 28.20 | 3.75 | 49.3 | 11.62 |
| Sant Antoni de Portmany | BI | 39.03, 1.31 | 29 | No | 27.92 | 4.33 | 88.6 | 30.51 |
| Sant Joan de Labritja | BI | 39.09, 1.58 | 30 | No | 29.85 | 4.69 | 107.1 | 40.52 |

* In Serra, we sampled in two different periods (winter, 77 trees; and spring, 30 trees). The 30 trees were a sub-sample of the 77.

** Squirrels density estimates were based on the abundance of cone cores under the trees (result of squirrels feeding activity).

Table S2: Summary of models for a) DBH and b) fire proneness in relation to squirrel presence. DBH model was fitted at individual level, including site as random factor. Fire proneness model was fitted at site level. Estimated coefficients ± standard error (SE), *t* and *p* values are shown.

| Model | Estimate ± SE | *t* value | *p* value |
| --- | --- | --- | --- |
| **a)** **DBH (LMM)** |  |  |  |
| Intercept | 28.659 ± 1.769 | 16.203 | <0.001*** |
| Squirrel’s presence | -1.397 ± 2.486 | -0.562 | 0.603 |
| **b)** **Fire proneness (LM)** |  |  |  |
| Intercept | 81.67 ± 22.77 | 3.587 | 0.023 * |
| Squirrel’s presence | 11.23 ± 32.20 | 0.349 | 0.745 |

# Notes S1: Study species.

*Pinus halepensis* is a common and widespread serotinous tree species in the Mediterranean basin and is dominant in the eastern Iberian Peninsula (Quézel, 2000). Its level of serotiny is controlled by fire regimes (Hernandez-Serrano et al., 2013; 2014; Ne’eman et al., 2004); however, to what extent pre-dispersal seed predation modulates the serotiny level for a given fire regime remains to be evaluated.

European red squirrels (*Sciurus vulgaris*) are widely distributed in mature *P. halepensis* forests of the Iberian Peninsula (Purroy, 2017). They are efficient pre-dispersal seed predators of conifer cones and negatively affect the abundance of other *P. halepensis* pre-dispersal seed predators such as crossbills (Mezquida and Benkman, 2005). Consequently, this squirrel species is considered the main selective agent influencing cone evolution in *P. halepensis* populations of the Iberian Peninsula.

# Notes S2: Sampling procedure details.

In *P. halepensis,* we can distinguish cones of different ages and maturation states by their colour: first-year immature cones (green), second-year recent ripened cones (during spring season, orangish-brown), second-year ripened cones (after spring season, bright brown), and third year and older cones (grey; Fig S1). From the third to sixth cohort it is impossible to distinguish the age of the cone by its colour (they are all grey), but we approximated their age from the position on the branch (counting whorls; Guiote and Pausas, 2023; Lamont, 1985; Moya et al., 2007). To minimize errors, we grouped the grey cones into two categories: recent and old grey cones (approximately belonging to the third-fourth or the fifth-sixth year, respectively). Then, in the three sites where squirrels were present, we counted the cone cores on the ground (at the base of the pine) and classified them into cohorts according to their colour (first, second, or third onward cohorts). Like other squirrels, *S. vulgaris* removes whole cones from the branches and eats them in the canopy (Mezquida and Benkman, 2005; Moller, 1986), so cone remains (i.e., cone cores) fall down under the tree. This allows us to quantify the cones consumed by the squirrels for an individual tree (this is why we avoided trees with overlapping crowns). For all measures involving cone cores from the ground, grey cones are considered as one category (not discriminating between recent and old grey cones) as grey depredated cones cannot be aged.

Figure S1: Differences in pine cone colour depending on their maturation stage. (a) First-year green cones, (b) second-year orangish-brown cones, (c) second-year from summer onward, bright brown cones, and (d) third-year and older, grey cones.

Notes S3: Cone trait measurement details. First, we measured the maximum dimensions of the closed cones (length and width), calculated the length-to-width ratio (hereinafter cone shape), weighed them, and counted the number of scales along the vertical axis. We then placed the cones in an oven at 65°C, monitoring them to determine the time until the opening begins (hereinafter ‘cone opening duration’; the cones were checked at 30' and then every 15' until they opened). We left the cones in the oven for an additional 45’ after they opened and measured the maximum cone width again and calculated the difference between the width of the open and closed cone divided by the width of the closed cone as an estimate of the flexibility of the scales. More flexible scales form a greater angle with respect to the central axis of the cone, resulting in a greater difference in width. Then, we used the complementary of flexibility (1 - flexibility) as a variable to represent scale rigidity. Next, we put them back into the oven (this time at 80°) until they opened completely and we extracted all the seeds. Then, we counted the number of full and empty seeds (i.e., viable and non-viable seeds), and weighed five full seeds per cone (without their wings). We also measured the thickness and width of three scales in the middle part of the cone (longitudinally), approximately equidistant from each other (radially; Fig. S2), and calculated mean values for each cone.

Figure S2: Schematic representation of scale trait measured on pine cones. Coloured scales indicate the position of the three scales that were measured (in the middle part of the cone and approximately equidistant from each other. Zoom (a) represents scale width, and zoom (b) represents scale thickness.

Table S3: Details of the literature search study sites. Study id, geographical coordinates (Coordinates; latitude and longitude, ^o^), region id, number of sampled trees (N), mean tree height per site (Height), squirrel activity (squirrels; records/km^2^), fire proneness as the mean monthly precipitation amount of the warmest quarter (FP, kg m^-2^ month^-1^) and mean tree serotiny degree values per site (Serotiny; %).

| Study id | Coordinates | Region id | N | Height | Squirrels | FP | Serotiny |
| --- | --- | --- | --- | --- | --- | --- | --- |
|  |  |  |  |  |  |  |  |
| 1 | 39.12 ,-0.39 | IP | 40 | 9.4 | 0.00 | 48.0 | 54.21 |
|  | 40.10 ,0.04 | IP | 40 | 11.6 | 0.13 | 79.1 | 38.10 |
|  | 39.74, -0.48 | IP | 63 | 10.8 | 0.27 | 76.1 | 52.94 |
|  | 39.87, -0.29 | IP | 67 | 12.7 | 0.08 | 82.3 | 52.70 |
|  | 40.35, 0.32 | IP | 40 | 10.8 | 0.05 | 80.4 | 61.35 |
|  | 40.05, -0.59 | IP | 40 | 11.9 | 0.03 | 92.5 | 28.93 |
|  | 39.8, -1.20 | IP | 67 | 11.8 | 0.03 | 91.0 | 26.70 |
|  | 39.89, -1.30 | IP | 40 | 10.2 | 0.01 | 93.0 | 23.88 |
|  |  |  |  |  |  |  |  |
| 2 | 42.25, 2.78 | IP | 11 | 5.0 | 0.03 | 203.8 | 24.59 |
|  | 41.06, 0.76 | IP | 15 | 5.1 | 0.03 | 73.0 | 47.87 |
|  | 41.67, 1.76 | IP | 18 | 5.2 | 0.07 | 141.1 | 39.11 |
|  | 42.23, -0.96 | IP | 25 | 4.7 | 0.02 | 116.9 | 75.31 |
|  | 41.92, -0.92 | IP | 25 | 4.3 | 0.00 | 79.9 | 69.87 |
|  | 41.35, -1.05 | IP | 26 | 4.6 | 0.01 | 83.2 | 44.67 |
|  | 41.10, -0.42 | IP | 19 | 4.8 | 0.00 | 85.2 | 39.14 |
|  | 40.80, 0.03 | IP | 13 | 5.3 | 0.02 | 108.1 | 40.02 |
|  | 40.74, -2.39 | IP | 24 | 4.4 | 0.00 | 79.8 | 30.93 |
|  | 40.45, -2.87 | IP | 20 | 4.3 | 0.01 | 65.2 | 41.85 |
|  | 39.82, -1.16 | IP | 27 | 4.8 | 0.04 | 91.2 | 67.79 |
|  | 38.52, -0.65 | IP | 23 | 4.9 | 0.11 | 49.1 | 80.10 |
|  | 39.79, -0.61 | IP | 35 | 4.8 | 0.03 | 79.9 | 60.90 |
|  | 39.18, -1.25 | IP | 33 | 5.0 | 0.02 | 62.6 | 68.17 |
|  | 40.08, 0.03 | IP | 3 | 5.6 | 0.16 | 77.5 | 64.05 |
|  | 38.50, -0.30 | IP | 16 | 4.7 | 0.09 | 75.0 | 57.64 |
|  | 38.14, -1.43 | IP | 26 | 4.7 | 0.01 | 45.8 | 84.36 |
|  | 38.63, -2.28 | IP | 33 | 4.3 | 0.01 | 58.1 | 35.77 |
|  | 37.70, -2.74 | IP | 29 | 4.9 | 0.01 | 35.5 | 72.66 |
|  | 37.79, -2.02 | IP | 28 | 4.8 | 0.01 | 40.0 | 53.48 |
|  | 38.23, -2.47 | IP | 34 | 5.0 | 0.01 | 56.9 | 60.39 |
|  | 37.74, -3.15 | IP | 22 | 4.5 | 0.00 | 35.0 | 87.07 |
|  | 38.11, -2.79 | IP | 35 | 4.8 | 0.09 | 60.8 | 71.65 |
|  | 36.82, -3.69 | IP | 22 | 4.9 | 0.06 | 28.6 | 81.04 |
|  | 36.84, -4.83 | IP | 26 | 4.3 | 0.00 | 21.9 | 90.69 |
|  | 36.82, -3.92 | IP | 31 | 4.9 | 0.09 | 39.5 | 85.92 |
|  | 39.81, 2.88 | BI | 26 | 5.0 | 0.00 | 78.6 | 68.76 |
|  | 39.57, 2.65 | BI | 12 | 4.6 | 0.00 | 84.2 | 33.76 |
|  | 39.36, 3.13 | BI | 12 | 4.8 | 0.00 | 79.0 | 47.32 |
|  |  |  |  |  |  |  |  |
| 3 | 39.10, -0.85 | IP | 14 | 4.6 | 0.02 | 57.4 | 83.90 |
|  | 38.38, -0.96 | IP | 16 | 4.5 | 0.03 | 41.6 | 91.30 |
|  | 37.75, -3.02 | IP | 10 | 4.1 | 0.01 | 47.2 | 87.70 |
|  | 39.59, 2.49 | BI | 15 | 4.1 | 0.00 | 98.8 | 71.90 |
|  | 38.74, 23.51 | Gr | 14 | 4.8 | 0.00 | 45.0 | 98.50 |
|  | 40.09, 23.91 | Gr | 12 | 5.0 | 0.00 | 73.6 | 75.80 |
|  | 40.62, 17.12 | It | 11 | 4.2 | 0.00 | 81.0 | 77.90 |
|  | 35.56, 8.65 | Tu | 16 | 4.0 | 0.00 | 71.8 | 85.80 |
|  |  |  |  |  |  |  |  |
| 4 | 44.27, 5.22 | Fr | 29 | 9.6 | 0.21 | 166.3 | 15.93 |
|  | 44.27, 5.21 | Fr | 29 | 8.4 | 0.22 | 164.8 | 7.59 |
|  | 44.26, 5.21 | Fr | 30 | 8.5 | 0.22 | 164.6 | 3.64 |
|  | 43.68, 5.39 | Fr | 20 | 7.1 | 0.69 | 101.8 | 19.16 |
|  | 43.50, 5.58 | Fr | 19 | 8.2 | 0.86 | 84.8 | 3.42 |
|  | 43.39, 5.06 | Fr | 20 | 8.6 | 0.81 | 65.7 | 3.28 |
|  | 43.36, 5.55 | Fr | 20 | 7.9 | 1.18 | 90.8 | 0.53 |
|  | 43,19, 5.60 | Fr | 20 | 8.6 | 1.15 | 124.6 | 1.95 |
|  | 44.25, 5.29 | Fr | 30 | 8.1 | 0.19 | 163.3 | 0.66 |
|  | 44.26, 5.29 | Fr | 28 | 7.5 | 0.18 | 164.5 | 0.48 |
|  | 44.22, 5.33 | Fr | 29 | 8.1 | 0.27 | 164.6 | 3.26 |
|  | 43.70, 5.09 | Fr | 20 | 8.4 | 0.78 | 88.5 | 6.04 |
|  | 43.47, 5.50 | Fr | 20 | 9.5 | 1.06 | 79.9 | 25.59 |
|  | 43.46, 4.99 | Fr | 20 | 8.2 | 1.68 | 66.7 | 17.00 |
|  | 43.39, 5.57 | Fr | 20 | 8.5 | 1.06 | 91.5 | 1.08 |
|  | 43.24, 5.58 | Fr | 20 | 10.1 | 1.15 | 77.4 | 4.30 |
|  |  |  |  |  |  |  |  |

**Study id:** 1) Hernández‐Serrano et al., 2013; 2) Hernández‐Serrano et al., 2014; 3) Martín-Sanz et al., 2016; 4) Romero and Ganteaume, 2020. **Region id**: IP (Iberian Peninsula); BI (Balearic Island); Gr (Greece); It (Italy); Tu (Tunisia); Fr (France).

Notes S4: Details about the methods to test the effect of predation on serotiny degree at geographical scale. After conducting the bibliographic search, we excluded studies with only one sampled site, those reporting serotiny data as mean values per site (rather than per individual), or studies that did not measure tree height or diameter at breast height (DBH). For Hernández‐Serrano et al. (2013) study, which reported DBH instead of height like the other studies, we performed a diameter-height allometry to estimate the tree heights. To achieve this, we used the Forest Explorer database (available at forestexplorer.gsic.uva.es), and selected the 100 *Pinus halepensis* trees closest to each of the sampled sites (using a distance matrix between the central points of the sampled sites and all data in the Forest Explorer database). We then fitted a linear regression model for each data set (corresponding to each sampled site) and used the intercepts and coefficients from these models to estimate the height of each tree based on its DBH.

Two studies were performed in common gardens and shared some of the population's provenances (Hernández‐Serrano et al. 2014, and Martín-Sanz et al. 2016). Therefore, we used the data included in Hernández‐Serrano et al. 2014 (with a more complete trial) and only used the data from Martín-Sanz et al. 2016 of the missing provenances. Since Martín-Sanz et al. (2016) reported serotiny data estimated in three common gardens, we sought to find the most similar environmental conditions to those in Hernández-Serrano et al. (2014). To do this, we estimated the annual growth rate of the pines from the shared provenances in each common garden and selected the site with the annual growth rate most similar to that reported in Hernández-Serrano et al. (2014) to obtain the missing provenance data (absolute differences in growth rate between common gardens averaged 2.2 cm).

To estimate the activity of squirrels in each studied site, we first obtained all available records of *Sciurus vulgaris* and then filtered out fossil and preserved specimens. Later, after establishing the influence area around each studied site, and given that some were located near the sea or inland waters, we corrected the area to account for only the land percentage. These values were what we used to calculate squirrel activity (squirrels’ records/km^2^ of land).

In all the studies considered, the serotiny degree was estimated by dividing the number of closed cones by the total number of cones, excluding the two most recent cohorts (immature cones).

Table S4: Climatic variables included in the model to test the influence of squirrel’s presence in explaining seed production per cone. The variables included in the model and an explanation of them is included. Climatic variables were obtained from the CHELSA V.2.x database (a gridded map 0.0083° resolution from average values from the 1981-2010 period) of the Swiss Federal Research Institute (Karger et al. 2021).

| Variable | Explanation |
| --- | --- |
| Annual precipitation  amount (kg m^-2^ year^-1^) | Accumulated precipitation amount over 1 year |
| Mean monthly precipitation amount of the wettest quarter (kg m^-2^ month^-1^) | The wettest quarter of the year is determined (to the nearest month) |
| Mean monthly precipitation amount of the driest quarter (kg m^-2^ month^-1^) | The driest quarter of the year is determined (to the nearest month) |
| Mean monthly precipitation amount of the warmest quarter (kg m^-2^ month^-1^) | The warmest quarter of the year is determined (to the nearest month) |
| Mean monthly precipitation amount of the coldest quarter (kg m^-2^ month^-1^) | The coldest quarter of the year is determined (to the nearest month) |
| Mean monthly climate moisture index (kg m^-2^ month^-1^) | Average monthly climate moisture  index over 1 year |
| Annual range of monthly climate moisture index (kg m^-2^ month^-1^) | Difference between maximum and minimum monthly climate moisture index |

Table S5: Summary of pairwise linear regression testing the influence of cone traits and serotiny in relation to predation degree of individual trees in each Serra sampling a) winter and b) spring). Estimated coefficients ± standard error (SE), *z* and *p* values are shown.

| Trait | Estimate ± SE | *z* value | *p* value |
| --- | --- | --- | --- |
| **a)** **Winter sampling** |  |  |  |
| Cone length (mm) | -0.004 ± 0.018 | -0.249 | 0.803 |
| Cone width (mm) | -0.063 ± 0.055 | -1.135 | 0.256 |
| Cone shape | 0.544 ± 0.823 | 0.676 | 0.508 |
| Number of scales | 0.064 ± 0.103 | 0.632 | 0.528 |
| Scale thickness (mm) | -0.080 ± 0.435 | -0.183 | 0.855 |
| Scale width (mm) | -0.113 ± 0.156 | -0.725 | 0.469 |
| Cone mass (g) | -0.025 ± 0.026 | -0.953 | 0.340 |
| Number of full seeds | 0.000 ± 0.012 | 0.019 | 0.985 |
| Number of empty seeds | -0.002 ±0.125 | -0.064 | 0.949 |
| Number of total seeds | 0.000 ± 0.011 | -0.011 | 0.991 |
| Seed mass (g) | -0.800 ± 0.666 | -1.201 | 0.230 |
| Seed-to-cone mass ratio | -0.808 ± 22.204 | -0.036 | 0.971 |
| Scale rigidity | -0.577 ± 1.737 | 0.332 | 0.740 |
| Cone opening duration (min) | 0.001 ± 0.002 | 0.478 | 0.632 |
| Pre-predation serotiny degree | 1.901 ± 0.464 | 4.095 | <0.001*** |
| **b)** **Spring sampling** |  |  |  |
| Cone length (mm) | 0.004 ± 0.023 | 0.171 | 0.865 |
| Cone width (mm) | -0.009 ± 0.079 | -0.114 | 0.909 |
| Cone shape | 0.315 ± 1.215 | 0.259 | 0.796 |
| Number of scales | -0.004 ± 0.148 | -0.030 | 0.976 |
| Scale thickness (mm) | -0.307 ± 0.927 | -0.331 | 0.740 |
| Scale width (mm) | 0.009 ± 0.186 | 0.050 | 0.960 |
| Cone mass (g) | -0.003 ± 0.034 | -0.075 | 0.940 |
| Number of full seeds | 0.010 ± 0.026 | 0.366 | 0.714 |
| Number of empty seeds | 0. 091 ± 0.096 | 0.947 | 0.344 |
| Number of total seeds | 0.013 ± 0.022 | 0.553 | 0.580 |
| Seed mass (g) | 0.935 ± 1.183 | 0.791 | 0.429 |
| Seed-to-cone mass ratio | 0.471 ± 0.240 | 1.962 | 0.049 * |
| Scale rigidity | -0.758 ± 0.227 | 3.335 | <0.001*** |
| Cone opening duration (min) | -0.338 ± 0.199 | -1.702 | 0.088 |
| Pre-predation serotiny degree | -0.381 ± 0.243 | -1.568 | 0.117 |

Table S6: Results of the model for serotiny degree considering all grey cone cohorts in relation to squirrels' presence and covariates (sequential addition of variables). Degree of freedom (df), Akaike information criterion (AIC), χ^2^ and the associated *p* value of each step in the model are presented. The last two columns provide the estimated coefficients and standard errors (SE) for the intercept (null model row) and fixed effects of the final model.

| Model | df | AIC | χ² | *p* | Estimate ± SE |
| --- | --- | --- | --- | --- | --- |
| Null | 3 | 1958.7 |  |  | 1.185 ± 0.534 |
| DBH | 4 | 1949.3 | 11.361 | <0.001*** | -0.048 ± 0.014 |
| + Squirrel’s presence | 5 | 1948.7 | 2.576 | 0.109 | -0.907 ± 0.510 |

Table S7: Results of model for serotiny degree (mean values per site) in relation to cumulative burned area (CBA; cumulative burned area was estimated as the sum of fire sizes in a 10 km buffer around the sites, following the methodology described in Guiote and Pausas, 2023). We fit a generalized linear mixed model (GLMM) with a Betabinomial error distribution and the logit link function. CBA was tested against null model. Degree of freedom (df), Akaike information criterion (AIC), χ^2^ and the associated *p* value are presented.

| Model | df | AIC | χ² | *p* value |
| --- | --- | --- | --- | --- |
| Null | 3 | 1383.1 |  |  |
| CBA | 4 | 1383.6 | 1.497 | 0.221 |

# Table S8: Mean cone measurements of the six sampled sites and summary of the models (linear mixed models including site as random factor) testing the influence of squirrel’s presence in each cone trait (*t* and *p*-values are shown).

|  | **Squirrels present** | | |  | **Squirrels absent** | | |  |  |
| --- | --- | --- | --- | --- | --- | --- | --- | --- | --- |
| Trait | Serra | Artana | Vandellòs i l'Hospitalet de l'Infant |  | Llombay | Sant Antoni de Portmany | Sant Joan de Labritja | *t* value | *p* value |
| Cone length (mm) | 65.40 | 57.50 | 43.40 |  | 51.50 | 46.10 | 48.40 | 1.009 | 0.370 |
| Cone width (mm) | 30.80 | 29.50 | 25.10 |  | 26.80 | 23.80 | 24.10 | 1.748 | 0.155 |
| Cone shape | 2.11 | 1.95 | 1.72 |  | 1.91 | 1.92 | 2.00 | -0.131 | 0.902 |
| Number of scales | 11.20 | 12.10 | 10.00 |  | 9.76 | 9.21 | 10.70 | 1.627 | 0.179 |
| Scale thickness (mm) | 3.47 | 3.51 | 2.98 |  | 3.36 | 2.70 | 2.63 | 1.340 | 0.251 |
| Scale width (mm) | 12.30 | 10.80 | 10.10 |  | 10.70 | 9.40 | 9.12 | 1.647 | 0.175 |
| Cone mass (g) | 23.50 | 18.10 | 10.00 |  | 13.90 | 9.90 | 10.30 | 1.453 | 0.220 |
| Number of full seeds | 30.50 | 29.90 | 16.80 |  | 30.30 | 47.30 | 54.80 | 1.453 | 0.220 |
| Number of empty seeds | 6.75 | 9.39 | 8.07 |  | 9.79 | 18.30 | 20.00 | -2.605 | 0.060 |
| Number of total seeds | 37.30 | 39.30 | 24.90 |  | 40.10 | 65.60 | 74.80 | -2.149 | 0.098 |
| Seed mass (g) | 0.02 | 0.02 | 0.01 |  | 0.02 | 0.01 | 0.01 | 0.285 | 0.285 |
| Seed-to-cone mass ratio | 0.03 | 0.02 | 0.02 |  | 0.04 | 0.04 | 0.04 | -4.171 | 0.014 * |
| Scale rigidity | 0.33 | 0.27 | 0.22 |  | 0.32 | 0.34 | 0.32 | 0.233 | 0.827 |
| Cone opening duration (min) | 114.00 | 109.00 | 69.80 |  | 159.00 | 87.50 | 49.60 | -0.143 | 0.893 |

Table S9: Summary of the models for a) canopy seed bank estimated holding the seed per cone constant, and b) canopy seed bank estimated holding the number of cones constant in relation to squirrel presence (considering annual crop size). Model a) represents the effect of squirrel’s presence on cone number, while model b) the effect on seeds per cone. Estimate ± SE, *z* and *p*- values are shown.

| Model | Estimate ± SE | *z* value | *p* value |
| --- | --- | --- | --- |
| **a) Effect on canopy seed bank** |  |  |  |
| Intercept | 8.176 ± 0.136 | 60.20 | <0.001*** |
| Annual cone production | 0.012 ± 0.002 | 7.53 | <0.001*** |
| Squirrel presence | -0.578 ± 0.157 | -3.670 | <0.001*** |
| **b) Effect on seeds per cone** |  |  |  |
| Intercept | 8.566 ± 0.145 | 59.26 | <0.001*** |
| Annual cone production | 0.003 ± 0.001 | 1.97 | 0.049 * |
| Squirrel presence | -0.468 ± 0.187 | -2.51 | 0.012 * |
|  |  |  |  |

Figure S3: Effect of squirrel’s presence on the two components of the canopy seed bank, cone number and seeds per cone. Effect size over each trait (yellow diamonds; left) and predicted values and confident intervals of models testing the influence of squirrel’s presence (“No” in blue, “Yes” in orange; right) holding the seeds per cone constant (i.e. the effect of squirrels on cone number; top) and holding the cone number constant (i.e. the effect of squirrels on seeds per cone; bottom).

Table S10: Summary of pairwise linear regression testing the relationship of cone traits and serotiny (arcsine-transformed) across all populations. Estimated coefficients ± standard error (SE), *t* and *p* values are shown.

| Trait | Estimate ± SE | *t* value | *p* value |
| --- | --- | --- | --- |
| Cone length (mm) | 0.003 ± 0.003 | 1.237 | 0.273 |
| Cone width (mm) | 0.007 ± 0.008 | 0.863 | 0. 389 |
| Cone shape | 0.164 ± 0.136 | 1.204 | 0.230 |
| Number of scales | 0.034 ± 0.020 | 1.720 | 0.086 |
| Scale thickness (mm) | 0.098 ± 0.077 | 1.263 | 0.208 |
| Scale width (mm) | 0.000 ± 0.000 | 0.300 | 0.764 |
| Cone mass (g) | 0.004 ± 0.004 | 0.837 | 0.404 |
| Number of full seeds | 0.002 ± 0.002 | 1.412 | 0.160 |
| Number of empty seeds | 0.000 ± 0.003 | 0.137 | 0.891 |
| Number of total seeds | 0.002 ± 0.002 | 1.630 | 0.106 |
| Seed mass (g) | 0.052 ± 0.097 | 0.537 | 0.592 |
| Seed-to-cone mass ratio | -0.324 ± 2.166 | -0.149 | 0.881 |
| Scale rigidity | -0.800 ± 0.277 | -2.883 | 0.004** |
| Cone opening duration (min) | 0.003 ± 0.000 | 7.905 | <0.001 *** |

Table S11: Results of the model for serotiny degree (arcsine-transformed) in relation to cone traits (sequential addition of variables). Degree of freedom (df), Akaike information criterion (AIC), χ^2^ and the associated p value of each step in the model are presented. The last two columns provide the estimated coefficients and standard errors (SE) for the intercept (null model row) and fixed effects of the final model.

| Model | df | AIC | χ² | *p* | Estimate ± SE |
| --- | --- | --- | --- | --- | --- |
| Null | 3 | 153.41 |  |  |  |
| Cone opening duration | 4 | 102.08 | 53.329 | <0.001*** | 0.003 ± 0.00 |

# Table S12: Results of the zero-inflated Gaussian mixed model testing the association between serotiny and seed-to-cone mass ratio in relation to squirrel presence.

| Model | Estimate ± SE | *z* value | *p* value |
| --- | --- | --- | --- |
| **Conditional model:** |  |  |  |
| Intercept | -1.559 ± 0.215 | -7.259 | <0.001*** |
| + Cone opening duration | 0.362 ± 0.041 | 8.872 | <0.001*** |
| Serotiny categorization | 1.798 ± 0.092 | 19.485 | <0.001*** |
| Seed-to-cone mass ratio | -0.023 ± 0.050 | - 0.462 | 0.644 |
| Squirrel’s presence | -1.246 ± 0.312 | -3.991 | <0.001*** |
| Serotiny categorization * seed-to-cone mass ratio | -0.054 ± 0.066 | -0.811 | 0.417 |
| Serotiny categorization * squirrel’s presence | 1.014 ± 0.204 | 4.977 | <0.001*** |
| Seed-to-cone mass ratio * squirrel’s presence | 0.126 ± 0.136 | 0.934 | 0.350 |
| Serotiny categorization* seed-to-cone mass ratio * squirrel’s presence | -0.745 ± 0.238 | -3.135 | 0.002** |
|  |  |  |  |

Table S13: Results of the of the zero-inflated Gaussian mixed model testing serotiny variability among regions across the Mediterranean basin (geographical analysis) in relation to tree height, fire proneness, and squirrel activity. The models were fitted by sequential addition of variables. Degree of freedom (df), Akaike information criterion (AIC), χ^2,^ and the associated *p-value* of each step in the model are presented. The last two columns provide the estimated coefficients and standard errors (SE) for the intercept (null model row) and fixed effects of the final model.

| Model | df | AIC | χ² | *p* | Estimate | SE |
| --- | --- | --- | --- | --- | --- | --- |
| **Conditional model:** |  |  |  |  |  |  |
| Null | 5 | -202.93 |  |  | 1.012 | 0.075 |
| Tree height | 6 | -242.14 | 41.214 | <0.001*** | -0.033 | 0.005 |
| + Fire proneness | 7 | -258.56 | 18.421 | <0.001*** | 0.003 | 0.001 |
| + Squirrel activity | 8 | -263.32 | 6.7579 | 0.009** | -0.242 | 0.088 |

# References:

**Hernández-Serrano A, Verdú M, González-Martínez SC, Pausas JG**. **2013**. Fire structures pine serotiny at different scales. *American Journal of Botany* **100**: 2349–2356. <https://doi.org/10.3732/ajb.1300182>.

**Hernández-Serrano A, Verdú M, Santos-del-Blanco L, Climent J, González-Martínez SC, Pausas JG**. **2014**. Heritability and quantitative genetic divergence of serotiny, a fire-persistence plant trait. *Annals of Botany* **114**: 571–577. <https://doi.org/10.1093/aob/mcu142>.

**Karger DN, Wilson AM, Mahony C, Zimmermann NE, Jetz W**. **2021**. Global daily 1 km land surface precipitation based on cloud cover-informed downscaling. *Scientific Data* **8**: 307. <https://doi.org/10.1038/s41597-021-01084-6>.

**Martín-Sanz RC, Santos-del-Blanco L, Notivol E, Chambel MR, San-Martín R, Climent J**. **2016**. Disentangling plasticity of serotiny, a key adaptive trait in a Mediterranean conifer. *American Journal of Botany* **103**: 1582–1591. <https://doi.org/10.3732/ajb.1600199>.

**Ne’eman G, Goubitz S, Nathan R**. **2004**. Reproductive traits of Pinus halepensis in the light of fire – a critical review. *Plant Ecology* **171**: 69–79. <https://doi.org/10.1023/B:VEGE.0000029380.04821.99>.

**Purroy F J**. **2017**. *Ardilla roja - Sciurus vulgaris*. Enciclopedia Virtual de Los Vertebrados Españoles. <https://www.vertebradosibericos.org/mamiferos/scivul.html>. 25 Sep. 2024.

**Quézel P**. **2000**. Taxonomy and biogeography of Mediterranean pines (*Pinus halepensis* and *P. brutia*). In: *Ecology, Biogeography and Management of Pinus halepensis and P. brutia Ecosystems in the Mediterranean Basin*., Ne’eman, G. and Trabaud, L. (eds). Leiden,

**Romero, B., and Ganteaume, A. 2020.** Does recent fire activity impact fire-related traits of *Pinus halepensis* Mill. and *Pinus sylvestris* L. in the French Mediterranean area? Annals of Forest Science **77:**106. <https://doi.org/10.1007/s13595-020-01016-1>
